# Faunal Communities Are Invariant to Fragmentation in Experimental Seagrass Landscapes

**DOI:** 10.1371/journal.pone.0156550

**Published:** 2016-05-31

**Authors:** Jonathan S. Lefcheck, Scott R. Marion, Alfonso V. Lombana, Robert J. Orth

**Affiliations:** 1 Virginia Institute of Marine Science, The College of William & Mary, Gloucester Point, Virginia, 23062, United States of America; 2 Oregon Department of Fish & Wildlife, Marine Resources Program, Newport, Oregon, 97365, United States of America; Australian National University, AUSTRALIA

## Abstract

Human-driven habitat fragmentation is cited as one of the most pressing threats facing many coastal ecosystems today. Many experiments have explored the consequences of fragmentation on fauna in one foundational habitat, seagrass beds, but have either surveyed along a gradient of existing patchiness, used artificial materials to mimic a natural bed, or sampled over short timescales. Here, we describe faunal responses to constructed fragmented landscapes varying from 4–400 m^2^ in two transplant garden experiments incorporating live eelgrass (*Zostera marina* L.). In experiments replicated within two subestuaries of the Chesapeake Bay, USA across multiple seasons and non-consecutive years, we comprehensively censused mesopredators and epifaunal communities using complementary quantitative methods. We found that community properties, including abundance, species richness, Simpson and functional diversity, and composition were generally unaffected by the number of patches and the size of the landscape, or the intensity of sampling. Additionally, an index of competition based on species co-occurrences revealed no trends with increasing patch size, contrary to theoretical predictions. We extend conclusions concerning the invariance of animal communities to habitat fragmentation from small-scale observational surveys and artificial experiments to experiments conducted with actual living plants and at more realistic scales. Our findings are likely a consequence of the rapid life histories and high mobility of the organisms common to eelgrass beds, and have implications for both conservation and restoration, suggesting that even small patches can rapidly promote abundant and diverse faunal communities.

## Introduction

The alteration and destruction of nearshore ecosystems as the result of anthropogenic activities is second only to climate change in terms of potential negative impacts on the world’s coasts [1]. Fishing practices, coastal engineering, nutrient and sediment runoff, storms, disease, and invasive species have all been linked to the fragmentation and eventual loss of coastal habitats [2]. Among the most affected habitats are seagrasses, aquatic angiosperms that are distributed worldwide. Globally, seagrass losses have been accelerating from a median of 0.9% year^-1^ before 1940 to 7% year^-1^ since 1990 [3], and this decline may impair a number of essential services, including nursery functions, sediment stabilization and shoreline buffering, nutrient cycling, and carbon storage [4]. Since many seagrasses have superficial root systems and are common in shallow subtidal areas that are subject to intense development [5], they are particularly vulnerable to disturbance and fragmentation [3].

Recent syntheses, however, have found overwhelmingly null [6], or equally contrasting positive and negative [7], consequences of fragmentation for the associated animal communities. Abundance, diversity, and other faunal community characteristics are generally higher in seagrass than adjacent vegetated areas [8,9], but so far appear to be invariant to the patchiness of habitat, instead responding more strongly to the presence and amount of aboveground biomass [7,10]. A recent review suggested that the prevalence of non-significant fragmentation effects in seagrasses may be an artifact of experimental designs, where patch size is confounded with other environmental gradients such as habitat complexity, depth, patch shape, position along the coast, and low replication, all of which act to increase sampling variation [6]. Another review also note that most studies do not consider temporal dynamics [7], surprising since fragmentation is by definition the *process* of breaking apart, further complicating the issue (but see [11,12]).

Despite the lack of empirical consensus, there have been a number of hypotheses proposed to explain the potential effects of seagrass fragmentation on associated fauna (reviewed in [6]). Foremost is the idea that predation is higher in fragmented habitats, owing to increased foraging efficiency in reduced habitat, the concentration of prey at the edge of patches (i.e., increased resources), and greater access to the interior of (smaller) patches. Thus, predator abundance and diversity is expected to be higher in fragmented landscapes [13], whereas the opposite might be inferred for their prey. Finally, fragmentation is expected to increase the amount of edge habitat, increasing encounter rates, and leading to higher diversity and abundance in patchy landscapes [11]. This idea has been extended to the passive recruitment of planktonic larvae of nekton [14], and thus might reasonably apply to smaller and less mobile organisms whose juvenile or adult stages move with currents, such as peracarid crustaceans, decapods, and gastropods [15].

In addition to empirical evidence, theoretical models from terrestrial systems predict that fragmentation will actually increase diversity in the short term, with a decline in the longer term [16,17]. Fragmentation allows the dispersal of inferior competitors among an increasing number of patches, essentially allowing species to colonize a new patch before they have a chance to be driven to extinction in their current one [16]. The same disturbance that generates patchiness may also preferentially remove superior competitors, allowing inferior competitors to thrive in the absence of competition [16]. Ultimately, however, the continued and pervasive loss of habitat will drive local extinctions simply through reduction in habitat [17]. While the amount of habitat that needs to be lost to build this ‘extinction debt’ and the time scale over which it manifests may vary, the trend appears to be robust to both the duration and spatial configuration of the destruction. In other words, the loss of several smaller patches, whether immediate or gradual, should have the same effects on community diversity as the loss of one large patch of equivalent area [18], an idea which has some support in seagrass systems [14].

The design most often used for exploring seagrass fragmentation effects on faunal communities are surveys along a natural gradient of patchiness [19–26]. Surveys better reflect the actual spectrum and influence of patch size in nature. By design, however, such studies have difficulty assigning causation because they do not often account for other covariates. For example, structural complexity has been linked to higher abundance and diversity [27,28], but is rarely incorporated or addressed in studies of patch effects [7]. To control for these potentially confounding variables, a subset of experiments have employed artificial seagrass units (ASUs) that are deployed in or near natural beds [11,19,26,29,30]. This approach standardizes the size and complexity of patches, and is easier to sample. ASUs are, however, eponymously artificial, leading to concerns over whether animals respond similarly to artificial versus real habitat [6]. There are also constraints as to the size of ASUs that can be manipulated, typically on the scale of <1 m^2^ and no more than 20 m^2^ [6]. This is in contrast to surveys of natural patches, which have been up to 6000 m^2^ [31]. A compromise between the two is to experimentally modify existing habitat to generate patchiness, but to date, this approach has been adopted by only a single study [12].

Fragmentation studies in seagrass have generally focused on the size of individual patches, particularly the ratio of edge/interior habitat, but far fewer have stepped backed and explored how the degree of fragmentation (i.e., the number of fragments) in the landscape affects faunal communities (but see [11]). In this study, we report on two independent experiments that transplanted live eelgrass into fragmented landscapes ranging from 4–400 m^2^ total area to explore how the size of patchy landscapes affects associated animal communities, from fish and crab mesopredators to invertebrate grazers. By using live eelgrass, we avoid the potential artifacts associated with artificial substrates, and are able to vastly increase the scale of the experimental replicates to that approaching natural eelgrass beds. We repeated this design over four distinct sites in two different subestuaries of the Chesapeake Bay, USA and monitored the experiments over multiple time periods, allowing us to test predictions regarding the temporal effects of fragmentation and address concerns over poor spatial and environmental replication. We hypothesized that predator abundance and diversity would be higher per unit area with increasing landscape size, and oppositely, we expected prey community properties to decrease with increasing landscape size. Finally, we predicted that competitive interactions among predators would decline with increasing landscape size, and oppositely for their prey.

## Methods

To distinguish among the two experiments reported here, we refer to Experiment 1 (E1), and Experiment 2 (E2). Both experiments utilized the same general design, with minor modifications where noted.

### Study Sites

Experiment 1 was conducted in 1996–1997 at three sites in the lower Chesapeake Bay, USA: one in the York River Estuary (Site 1: 37.22 N, 76.48 W), and two in the James River Estuary (Site 2: 37.02 N, 76.35 W; Site 3: 37.02 N, 76.32 W). Although all three sites historically supported eelgrass, by the time of the experiments they contained no vegetation prior to the transplantation, owing to declining water quality and storm disturbance in the region [32]. The nearest bed to Site 1 was 5 km upriver, and 1 km for Sites 2 and 3. The water depth at the three sites ranged from 0.5–1.0 m at low tide, with a tidal amplitude of 0.7–1.0 m (neap and spring tides). Over the duration of the experiment, water temperatures ranged from 15–26°C, and salinity varied between 17–23 PSU [33].

Experiment 2 was conducted in 1998–1999 at two sites: the same site in the York River (E1, Site 1), and a different site in the James River (Site 4: 36.97 N, 76.40 W). The sites in the James River from Experiment 1 were not considered for use in this experiment because they had completely filled in during the intervening years, and we wished to limit the potential influences of nearby existing beds. The depth and tidal range at these sites were similar to E1. Over the duration of the sampling in 1999, water temperatures ranged from 15–28°C, and salinity between 16–23 PSU [34].

### Experimental Transplants

**Fig 1:**
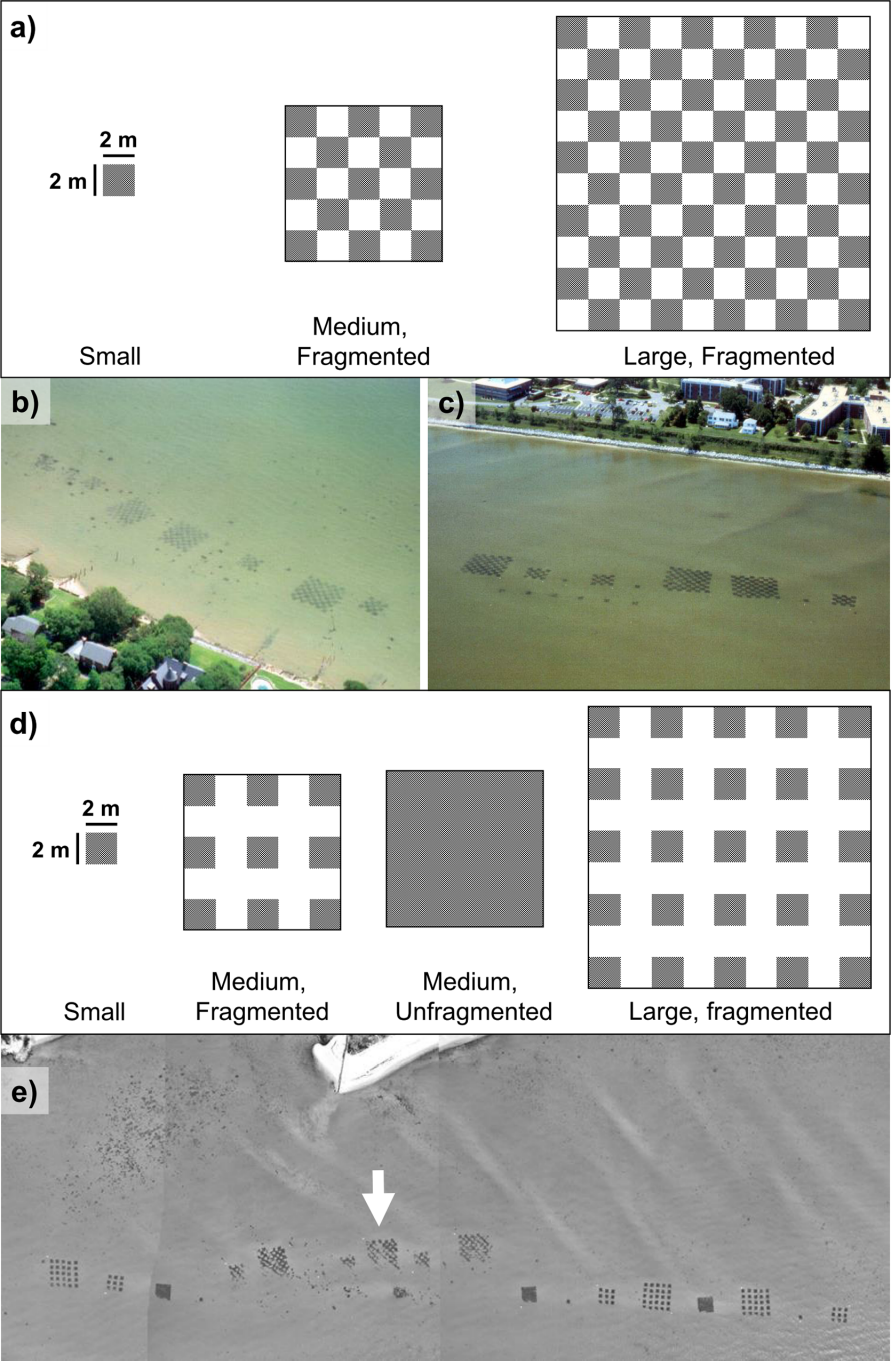
**(a)** A schematic of the experimental design employed in Experiment 1. Lower middle panels depict aerial photographs of transplants from Experiment 1 in the **(b)** James River Estuary, and (**c)** York River Estuary, taken June 1997 (approximately nine months after the initial planting) (from [35]). **(d)** A schematic of the experimental design employed in Experiment 1. Bottom panel depicts aerial photograph of transplants from Experiment 2 in the York River Estuary taken in June 1999 (approximately nine months after the initial planting). The arrow in panel **(e)** denotes a separate set of transplants conducted one-year prior for unrelated purposes, and is not reported on here.

In fall 1996 for E1, we generated three fragmented landscapes of differing sizes: small (4 m^2^), medium (100 m^2^), and large (400 m^2^). The medium and large landscapes consisted of thirdteen and fifty 4 m^2^ patches arranged in an alternating 5-x-5 and 10-x-10 m checkerboards, respectively(Fig 1A). Each treatment was replicated three times at each of the three sites for a total of nine replicates. Replicates were placed at least 10 m from one another to minimize disturbance and promote statistical independence. For each replicate, live eelgrass shoots were harvested from nearby beds and individual shoots were gently inserted 25–50 mm into bare sediment at the transplant sites based on the methods in [35]. Transplants were placed 15 cm from one another to generate the 4 m^2^ patches arranged corresponding to the treatments above.

In fall 1998 for E2, we generated four landscape types: small (4 m^2^), medium fragmented and unfragmented (both 100 m^2^), and large fragmented (324 m^2^) plots. The two larger fragmented treatments consisted of nine and twenty-five 4 m^2^ plots arranged in an alternating 5-x-5 and 9-x-9 m grids with 2 m buffers on the sides of every patch (Fig 1D). The slight alteration in the design was implemented to address the lack of results observed from E1, to explore whether a slight reconfiguration of the fragmented habitat might yield a different outcome. The medium unfragmented plot consisted of a single 10-x-10 m contiguous square of transplanted eelgrass containing the same total eelgrass area as the large fragmented plot, but with the same outer plot circumference as the medium fragmented plot, which allows for tests for effects of spatial arrangement while keeping total amount of habitat constant. Shoots were transplanted in equivalent densities and distances to E1, and each treatment was replicated three times at each of two sites for 6 total replicates. Replicates were placed at least 20 m from one another to once again discourage the potential for confounding interactions among replicates. In both experiments, replicates were placed parallel to the shoreline to minimize variation in depth.

### Plant Characteristics

To evaluate the effectiveness of the transplant procedure, percent cover and shoot density were assessed in the year following the initial transplantations. In summer and fall 1997 for E1, percent cover was assessed using a 2-*x*-2 m quadrat placed over four random transplanted patches in each treatment type, and the ratio of vegetated to unvegetated area was computed based on 12 categories ranging from 0–100% (based on the Braun-Blanquet crown cover scale, [36]). For shoot density, a 0.15 m^2^ metal ring was likewise placed over vegetated bottom and the number of individual shoots inside the ring were recorded. This procedure was conducted three times in each of the patches also censused for percent cover. In spring and fall 1999 for E2, percent cover was also assessed as above, but instead of shoot density, biomass cores were used to estimate dry weight of aboveground plant material in grams ash-free dry weight m^-2^ (see *Faunal Sampling*for more details about coring method).

### Faunal Sampling

Nekton, which we define as mobile benthic fauna, primarily decapod crustaceans and small fishes, such as pipefishes and blennies, were sampled in both experiments using a suction sampler. A weighted steel ring with a 1 mm mesh sleeve measuring 0.6 m in diameter was placed flush to the bottom at low tide, and the enclosed area was suctioned using a gasoline-powered pump for two minutes [33,37]. This method captures > 90% of mobile epibenthic and shallow benthic fauna < 10 cm in length or width, but not deeper burrowing infauna [33]. All animals were collected in a 0.8 mm mesh bag, placed on ice until unconscious, and returned to the lab and frozen. Samples were thawed at a later date, and all individuals enumerated to species.

In Experiment 1, suction samples were conducted in the growing season following the original transplants in July, September, October, and November of 1997. Each replicate of the small, medium, and large treatments was subsampled commensurate with their size: 1, 5, and 10 times, respectively. In the medium and large treatments, subsamples were taken from both the edge (outer ring of 2-x-2 m plots) and interior (>4 m from the edge) of the fragment. Within these two strata, subsamples were taken randomly for each replicate, treatment, and sampling date.

In Experiment 2, suction samples were taken at both the York and James River sites in June 1999, and in the James River in November 1999. The York River site was not resampled in November because many seagrass patches were lost as the result of storm-related wave damage in the intervening months. The small replicates were subsampled twice, the medium fragmented 8 times, the medium unfragmented 12–16 times, and the large fragmented 14–17 times. The variable sampling was a consequence of logistical constraints concerning the large number of samples taken during one specific tidal cycle. As in the first experiment, the placement of subsamples was randomly allocated evenly between interior and exterior portions of the medium and large treatments. We note that in both E1 and E2, there is only a single interior patch that is > 4m from the edge, so this patch was sampled consistently to ensure that the interior was always represented in the sampling pool.

Experiment 2 additionally collected core samples to quantify smaller, less mobile epifauna, primarily amphipods, isopods, and gastropods. This procedure consisted of placing a 13 cm diameter core tube flush with the bottom and using a sharp metal plate to sever the eelgrass at the sediment surface. The tube was inverted and its contents, including all grass and associated fauna, emptied into a 250 μm mesh bag. The core samples were treated identically to the suction samples, and all individuals enumerated to species. The eelgrass retained in the core tube was also separated, dried and weighed (see *Plant Characteristics*). Core samples were taken at the same time and in the general vicinity of all suction samples, with the exception of small (4 m^2^) plots, where four core samples but only two suction samples were taken, owing to the limit area available for sampling.

Vertebrates used in this study were sampled at a time before the College of William and Mary IACUC required institutional approval for the use of cold-blooded animals in research studies (ca. 2000). Thus, we were not required to undergo a formal review and authorization process, however animals were handled in a humane way to minimize potential suffering (euthanasia by ice bath immersion). All study sites were in public waters. Any member of the Virginia Institute of Marine Science may, "take for scientific purposes, any fish, shellfish or marine organism from the waters of Virginia" under the Commonwealth of Virginia Code 28.2-1101B. No endangered or protected species or locations were involved in this study.

### Community Responses

For each sample in each experiment, we calculated the following community responses: total abundance, species richness, evenness, Simpson diversity, and functional trait diversity (both presence-absence and abundance-weighted). Evenness was calculated as Pielou’s evenness *J* using the formula from [38]:

$$J=\frac{ln(D)}{ln(S)}$$ (1)

where *D* is Simpson diversity, and *S* is species richness. In this index, the lower limit of *J* is set at 0 (complete dominance by one species), and the upper limit at 1 (equal abundances across all species). Simpson diversity was first transformed into Hill numbers using the following equation [38]:

$$D=\frac{1}{1-D}$$ (2)

such that the units are interpreted as the effective number of species if all species were equally abundant. This transformation puts Simpson diversity into similar units as species richness, allowing direct comparison of the two. As *D* approaches *S*, individuals are distributed more equally among the suite of species.

For functional trait diversity, we scored seven functional traits that have been shown previously to discriminate among fauna of eelgrass beds [39,40]: exoskeleton material, trophic level, diet, body size (maximum length), mobility, reproductive mode (brooding vs. broadcast spawner), and development mode (planktonic larvae vs. direct development). These traits are also directly linked to the hypotheses concerning resource use (trophic level) and dispersal ability (mobility, reproductive and development mode) invoked to explain community responses to fragmentation. Further description of the traits and their ecological interpretation relative to fragmentation can be found in S1 Table, and raw trait values are provided in S2 Table.

To calculate functional diversity, we used the index of Rao’s quadratic entropy [41]:

$$Q=\sum_{i=1}^{S-1}\sum_{j=1}^{S}d_{ij}p_{i}p_{j}$$ (3)

where *d*_*ij*_ is the functional distance between species *i* and *j*, and *p* is their respective relative abundances. Functional distances were derived from a Gower dissimilarity matrix that incorporates both continuous (e.g., body size) and categorical (e.g., diet) traits into a single continuous measure of dissimilarity [42]. Because Gower’s distances are not ultrametric by default, and this property can lead to values of *Q* that are paradoxically maximized when fewer than all functional types are present [43], we converted these distances to ultrametric using the procedures in [44,45] before calculating *Q*. We imposed the transformation in Eq (2) to convert functional diversity into units of effective numbers of maximally distinct and equally abundant species. Finally, we calculated *Q* using presence-absence instead of relative abundance to down-weight the influence of highly abundant species, which yields a functional equivalent of richness.

### Statistical Analyses

We collapsed each response to mean values across all subsamples within a given replicate, date, and experiment. This was necessary to reduce potential bias due to the unavoidably uneven sampling effort employed across landscapes of dramatically differing size [40]. Because the community properties under investigation are known to scale with sampling effort according to a power law [46], we modeled each summarized response as a function of its sample size using a log-log relationship in all analyses. We also expressed all response variables in units m^-2^, which enables a fairer comparison between treatments of differing area. Together, these actions should alleviate the confounding effects of increasing patch size and sampling effort.

We employed general linear mixed effects models (GLMMs) to statistically test for the effect of landscape size and/or patchiness on each of the log-transformed responses. In addition to the log of the sample size, we also modeled landscape size, month, and their interaction as fixed effects. We chose to treat landscape size as continuous (i.e., m^2^) rather than categorical (i.e., small, medium, large) to minimize the degrees of freedom needed to estimate the models. We allowed the intercept of the fixed effects to vary by site and replicate, accounting for covariance among replicates within a site, as well as between sites. Model assumptions, including normality of errors and heteroscedasticity of variances, were assessed visually. All models were constructed using the *nlme* package [47].

Because we employed a repeated measures design, we tested different autocorrelation structures, but found they did not increase the likelihood of the models, based on comparison of AIC scores. Thus, we treated each time point as independent and uncorrelated in the final GLMMs. Significance was tested through Analysis of Deviance and Type III sums-of-squares using the *car* package [48], because we are explicitly interested in how the degree of fragmentation (landscape size) changed through time (month), thus emphasizing the significance of their interaction [49]. Because raw means would not account for differences in sampling effort addressed in the models, we additionally generated model-estimated (or marginal) means and standard errors from the GLMMs using the *effects* package [50], and binned these based on the initial treatment levels. We present the marginal means alongside the raw data points in the accompanying figures because they are more fairly compared.

To explore the consequences of fragmentation for potential competitive interactions, we employed the checkerboard or *C*-score, an index of co-occurrence that measures how often species are found together in the same treatment [51]. For each species pair, it is calculated as:

$${CS}_{ij}=(R_{i}-S)(R_{j}-S)$$ (4)

where *R* is the number of number of treatment replicates in which species *i* or *j* occurs, and *S* is the number of replicates that contain both species [52]. The values are then averaged over the entirety of the replicate-by-species matrix, such that the larger the *C*-score in a treatment, the less often pairs of species are found together. Because all species are physiologically capable of occupying all experimental patches, we interpret a high *C*-score as evidence for competitive exclusion. The *C*-score has been used detect competition under a variety of scenarios, and has been shown to be robust to substantial noise in the data [51].

Because the *C*-score can be influenced by the number of species and replicates in the dataset, we constructed a standardized effect size (SES) based on 5,000 random permutations of the dataset (keeping the row and column sums fixed at their observed values, so called ‘fixed-fixed’ algorithm). The SES is simply the observed *C*-score minus the mean of the simulated *C*-scores derived from the 5,000 permutated matrices, divided by the standard deviation of the simulated *C*-scores [53]. SES values < -2 indicate significantly greater co-occurrence than suggested by chance, and > 2 indicate significantly less co-occurrence than suggested by chance (i.e., competitive exclusion). Values falling between –2 and 2 indicate random segregation of species throughout the landscape. We analyzed the *C*-scores using the same GLMM framework as above, including sample size, landscape size, month, and landscape*month interaction as fixed effects, and site as a random effort. We did not, however, model this response to a power function, since the models met all assumptions in lieu of this transformation.

Finally, to explore changes in species composition as a function of landscape size and through time, we analyzed each experiment using non-metric multidimensional scaling (NMDS). We applied a Wisconsin square-root transformation to downplay the influence of highly abundant species and generated 95% confidence ellipses to test for significant differences in community composition between treatments (indicated by non-overlap of ellipses). NMDS was conducted using the *vegan* package [54], and ellipses using the package *ggplot2* [55]. We repeated all of the above analyses for suction samples for both Experiments 1 and 2, as well as for core samples in Experiment 2. All analyses were conducted in R v. 3.2.5 [56], and all data and R code used to conduct the analyses are provided in S1 File.

## Results

### Plant Characteristics

The experimental treatments maintained their patchiness through the duration of both experiments (Fig 1B, 1C and 1E). Analysis of variance of log_10_-transformed percent cover data from quadrat surveys of Experiment 1 revealed no significant treatment-by-site-by-month interactions (0.21 < *P* < 0.81), indicating no systematic difference in the seagrass density within experimental landscapes for a given sampling date (S1 Fig). Shoot density was generally lower in the fall (S1 Fig), representing the natural senescence of eelgrass in this region in the late summer [57].

Analysis of variance of log_10_-transformed percent cover data from quadrat surveys in Experiment 2 revealed significant interactions between month and treatment (*P* = 0.01), and site and treatment (*P* = 0.02). The significant interactions appear to be driven by higher percent cover in medium unfragmented plots in the York River during the spring of 1999 (S2 Fig). However, analysis of variance of log_10_-transformed dry mass data from the core samples revealed no significant interactions (*P* = 0.45 for month*treatment, and *P* = 0.53 for site*treatment). Since dry mass is a less subjective assessment of shoot density than percent cover, we have chosen to proceed with the full dataset, keeping this potential bias in mind. However, we note that the random structure of our models should account for some of this bias based on the association of shoot density with both replicate (treatment) and site.

### Experiment 1: Nekton (Suction Samples)

**Fig 2:**
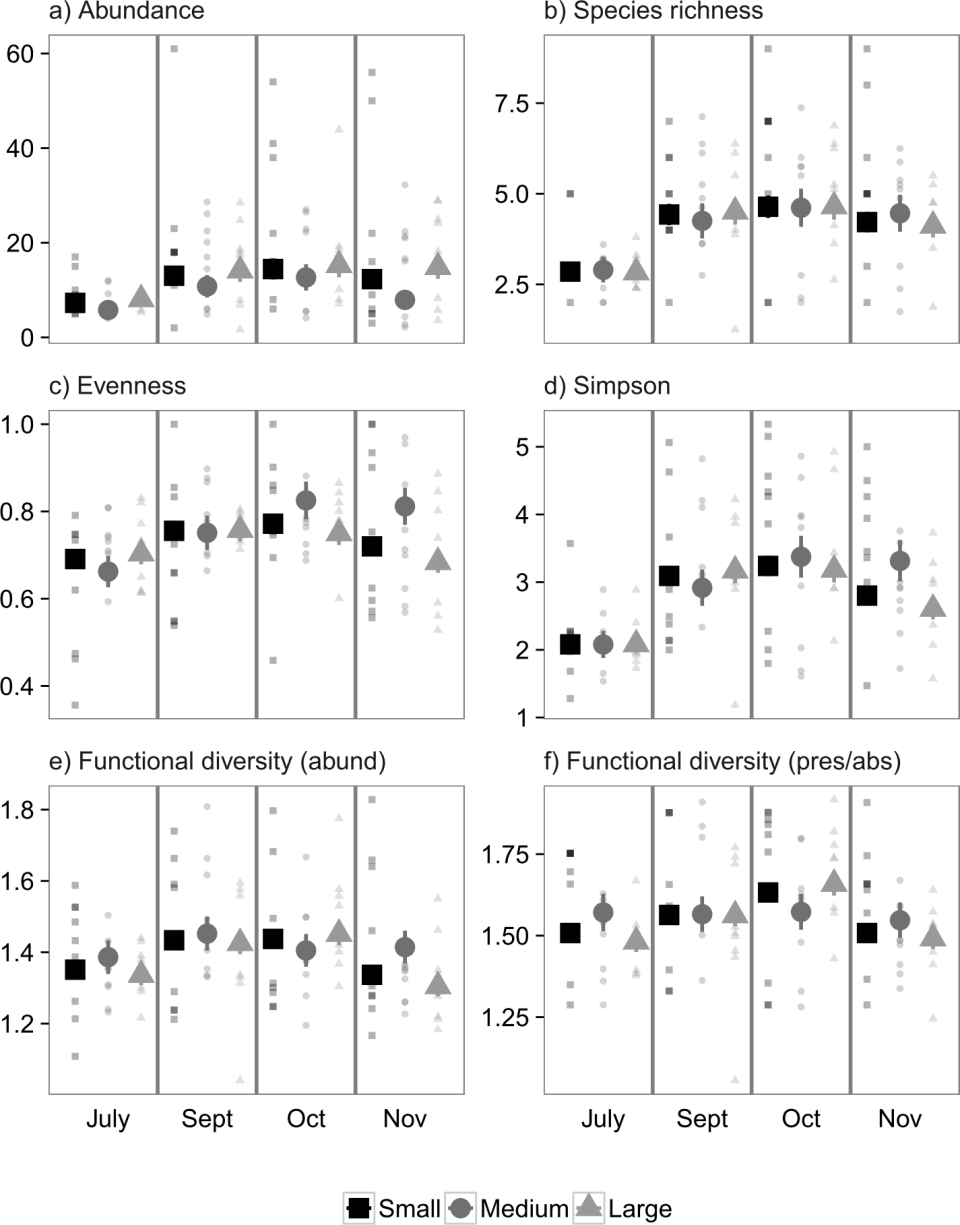
Mean values ± 1 SE m^-2^ for community properties obtained from suction sampling of nekton in Experiment 1. Colors and shapes correspond to small (4 m^2^), medium (100 m^2^), and large (400 m^2^) fragmented experimental landscapes of transplanted eelgrass. Small points correspond to replicate values. Large points are marginal means ± 1 SE estimated from generalized linear mixed effects models that account for variable sampling effort.

For suction-sampled nekton in Experiment 1, there was no significant effect of the experimental treatments on any of the six community metrics (0.58 < *P* < 1, Fig 2). There were, however, significant effects of month on evenness (*P* = 0.009, Fig 2C) and Simpson diversity (*P* = 0.006, Fig 2D), with both increasing throughout the year. There were not, however, any significant interactions between landscape size and month (0.07 < *P* < 0.93), indicating that the temporal changes were not contingent on the experimental treatments.

**Fig 3:**
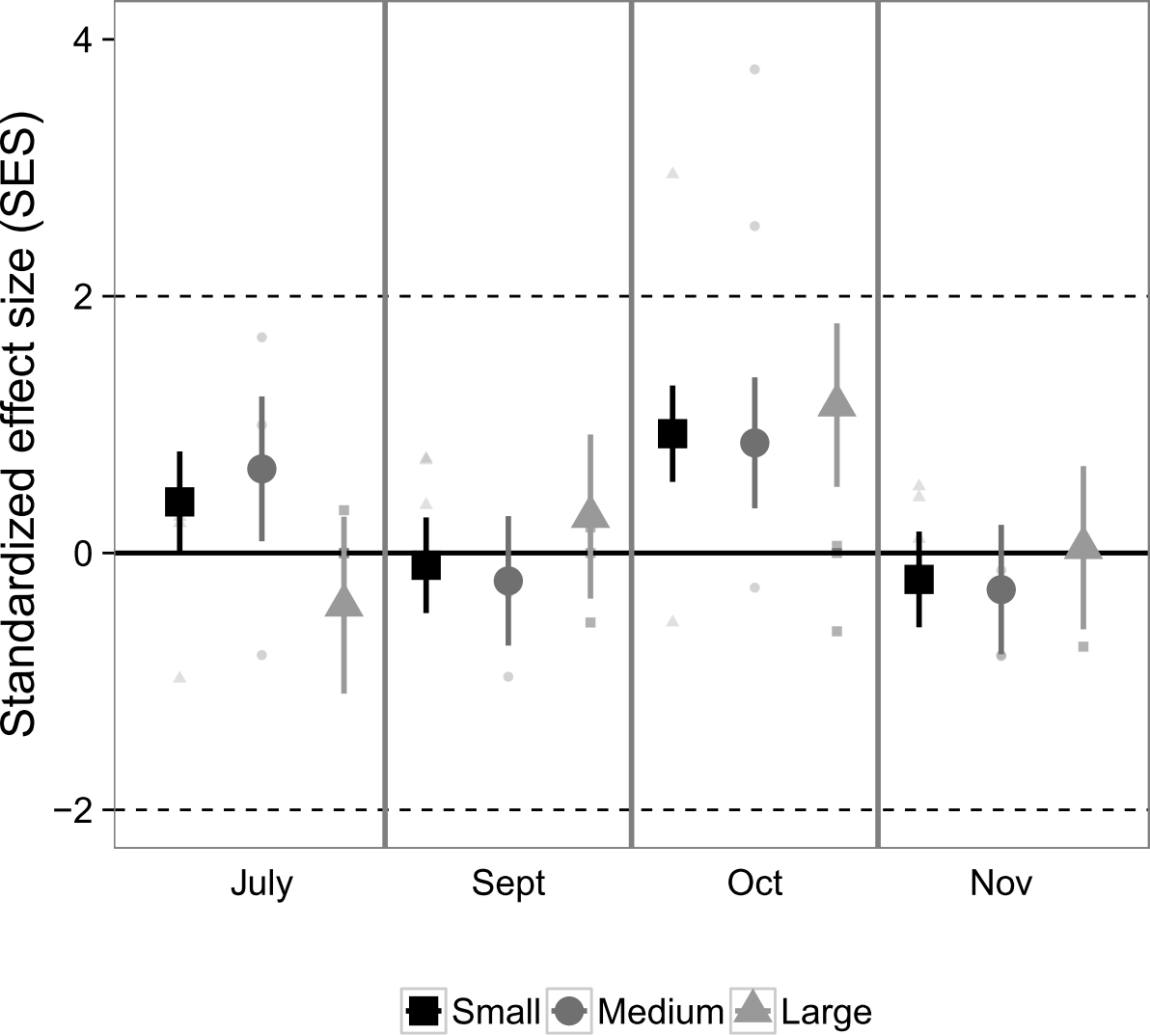
Standardized effect size (SES) from checkerboard scores, an index of nekton species co-occurrences in suction samples from Experiment 1. Values >2 indicate significantly fewer associations than would be expected from chance, whereas values < -2 indicate the opposite. Values in the range [-2, 2] indicate random segregation of species in experimental replicates. Large points are marginal means ± 1 SE estimated from generalized linear mixed effects models that account for variable sampling effort. Colors correspond to the size of the fragmented experimental landscape: small (4 m^2^), medium (100 m^2^), and large (400 m^2^).

Exploration of community co-occurrences revealed that species pairs, on average, appeared to be randomly segregated across the experimental landscapes, as indicated by the lack of standardized effect sizes falling outside of the [-2, 2] range (Fig 3), and was unaffected by landscape size (*P* = 0.33), sampling month (*P* = 0.26), or their interaction (*P* = 0.49), based on analysis of deviance.

**Fig 4:**
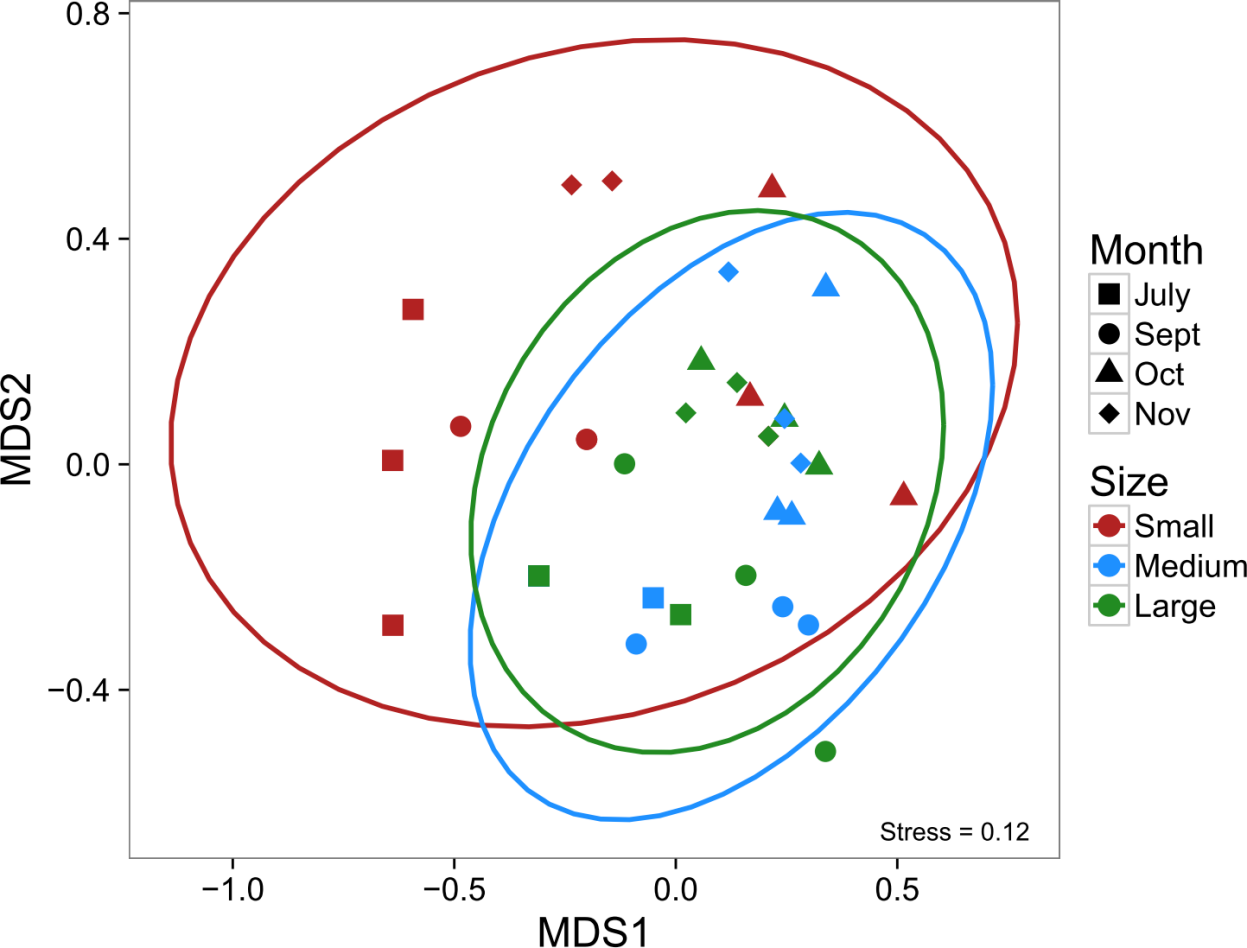
Non-metric multidimensional scaling of multivariate nekton community abundances from suction samples in Experiment 1. Colors correspond to the size of the fragmented experimental landscape: small (4 m^2^), medium (100 m^2^), and large (400 m^2^), and shapes to the month of sampling. Ovals are 95% confidence ellipses; overlap indicates that composition is not significantly different among the set of points based on α = 0.05.

Analysis of community composition using NMDS revealed no difference in composition among the three landscape sizes (as indicated by overlapping 95% confidence ellipses, Fig 4). There was, however, a tendency for small patches (4 m^2^) to have a more extreme community composition, which appeared to be driven principally by the grass shrimp (*Palaemonetes pugio*), mud crab (*Rhithropanopeus harissii*), and the pipefish (*Syngnathus* spp), particularly later in the year (S3 Fig). More apparent is the differences in community composition through time, a function of grass shrimp and broke-back shrimp (*Hippolyte pleuracanthus*) increasing in abundance during the fall (S3 Fig).

### Experiment 2: Nekton (Suction Samples)

**Fig 5:**
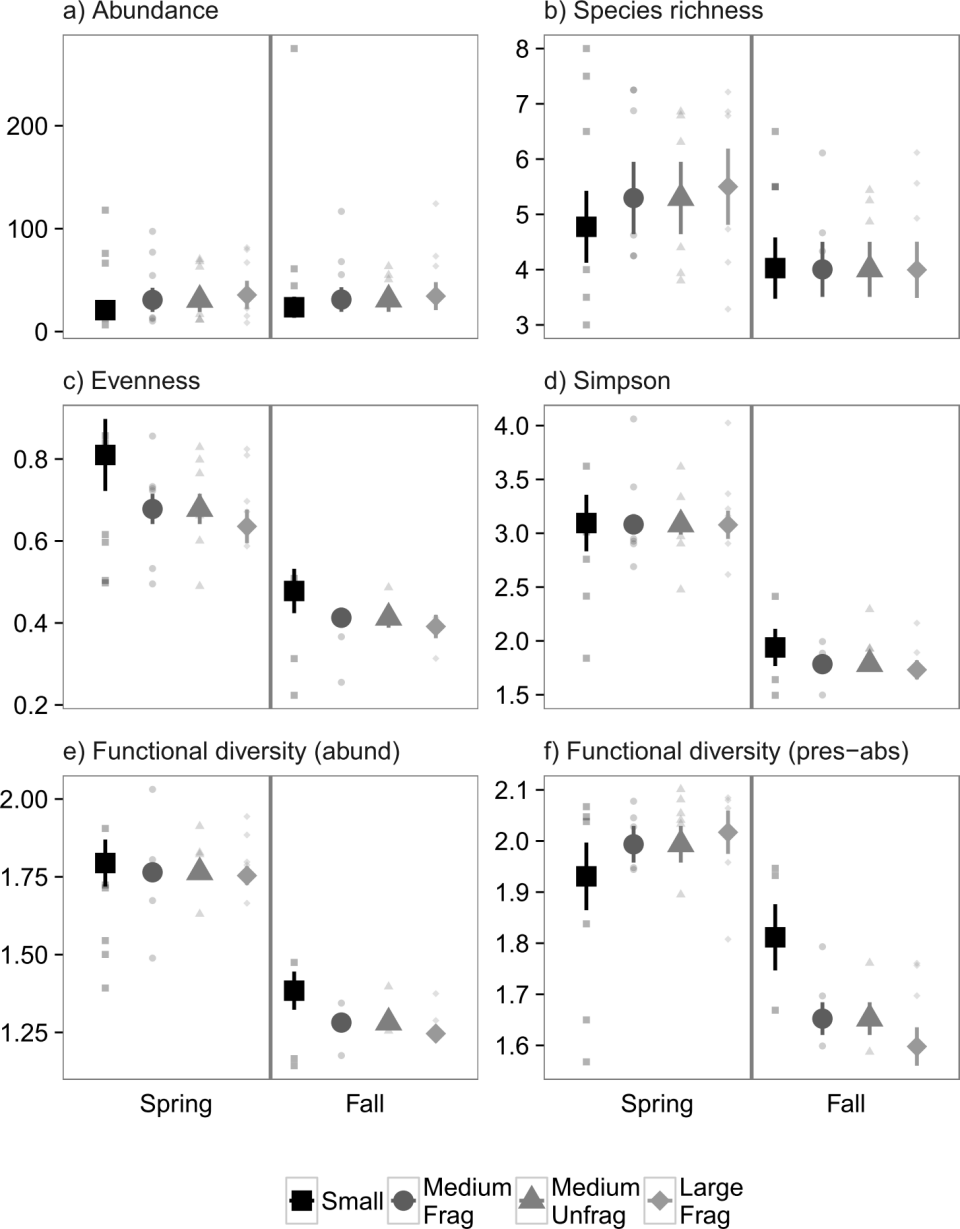
Mean values ± 1 SE m^-2^ for community properties obtained from suction sampling of nekton in Experiment 2. Colors and shapes correspond to small (4 m^2^), medium (100 m^2^), both fragmented and unfragmented, and large fragmented (400 m^2^) experimental landscapes of transplanted eelgrass. Small points correspond to replicate values. Large points are marginal means ± 1 SE estimated from generalized linear mixed effects models that account for variable sampling effort.

For suction-sampled nekton in Experiment 2, there was a significant month-by-treatment interaction for presence-absence weighted functional diversity (*P* = 0.002, Fig 5F). Exploration of parameter estimates revealed decreasing functional diversity with increasing landscape size later in the year (β = -0.04 ± 0.01). As with smaller fauna, however, there was no difference between the unfragmented medium and fragmented large landscapes (Fig 5F). There was no significant effect of landscape size (0.16 < *P* < 0.81) or the interaction between landscape size and month (0.38 < *P* < 0.98), for any of the remaining community responses (Fig 5A–5E). There was, however, an effect of season, with evenness (*P* = 0.002, Fig 5C), Simpson diversity (*P* = 0.004, Fig 5D), and abundance-weighted functional diversity (*P* = 0.003, Fig 5E) all significantly decreasing from the spring to the fall.

**Fig 6:**
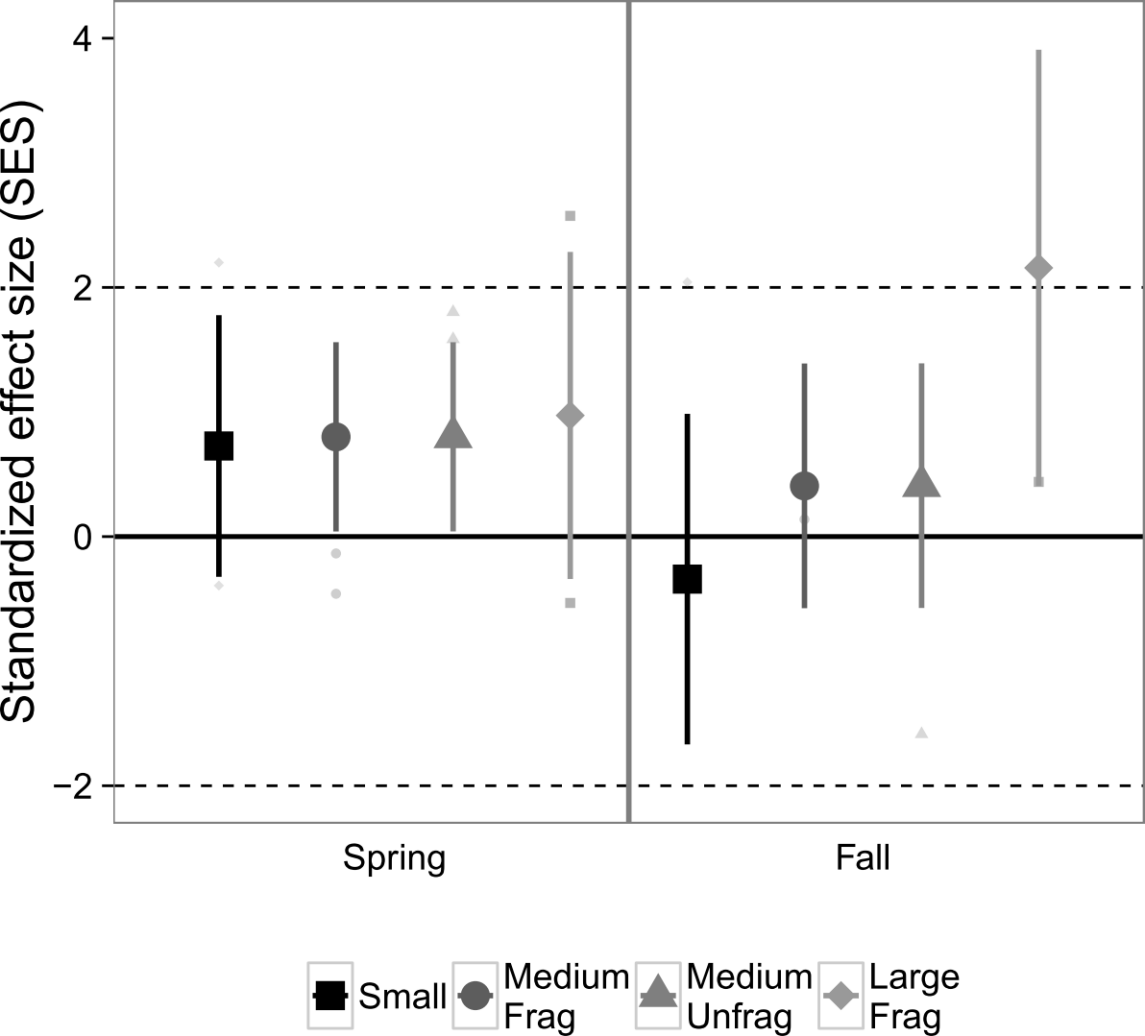
Standardized effect size (SES) from checkboard scores, an index of nekton species co-occurrences in suction samples from Experiment 2 across two seasons. Values >2 indicate significantly fewer associations than would be expected from chance, whereas values < -2 indicate the opposite. Values in the range [-2, 2] indicate random segregation of species in experimental replicates. Large points are marginal means ± 1 SE estimated from generalized linear mixed effects models that account for variable sampling effort. Colors correspond to the size of the experimental landscape: small (4 m^2^), medium (100 m^2^), both fragmented and unfragmented, and large fragmented (400 m^2^).

With respect to species co-occurrences and competition, there was no detectable deviation from randomness for nekton in Experiment 2 (Fig 6), nor any significant influences of landscape size (*P* = 0.30), month (*P* = 0.39), or their interaction (*P* = 0.38).

**Fig 7:**
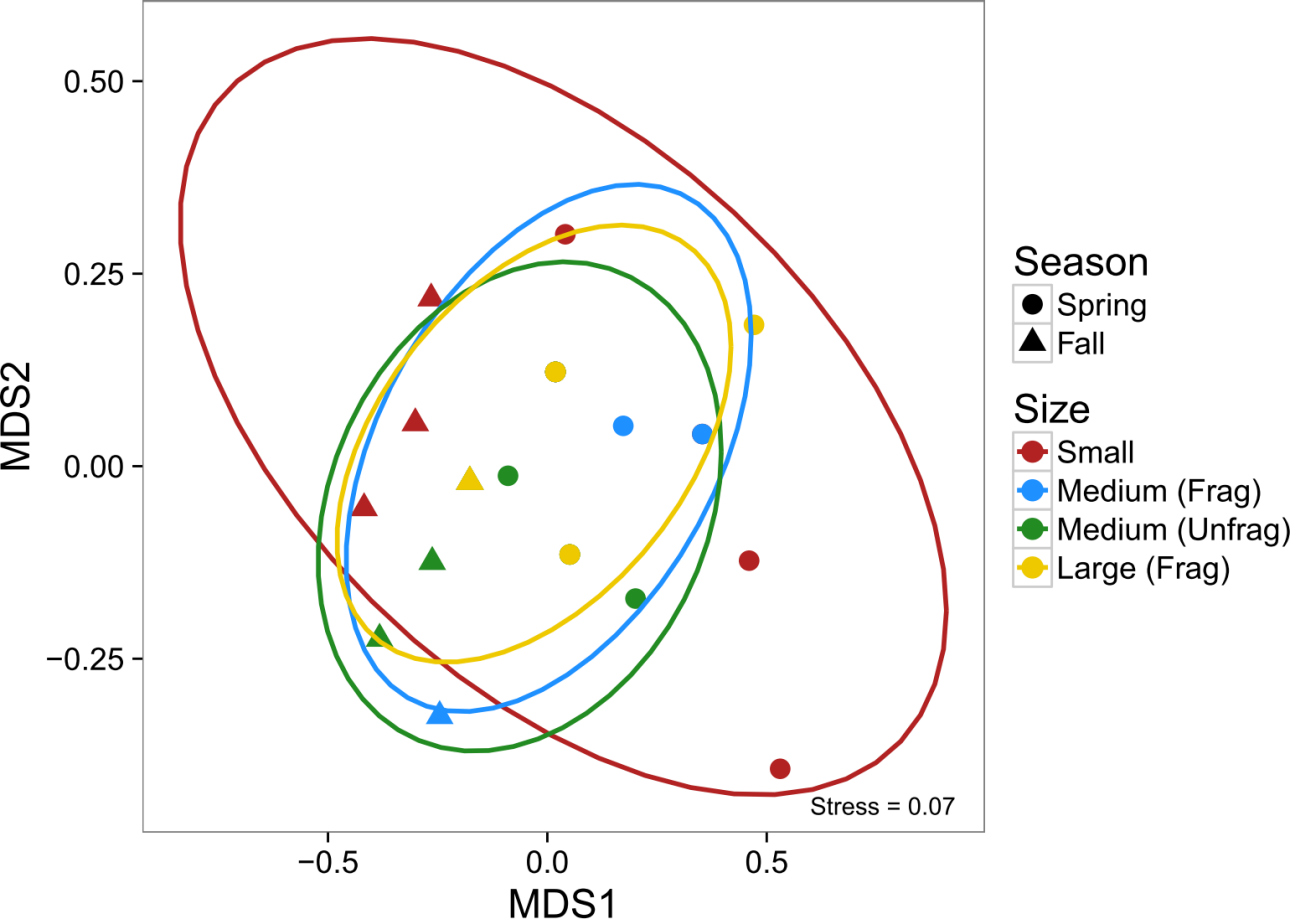
Non-metric multidimensional scaling of multivariate nekton community abundances from suction samples in Experiment 2. Colors correspond to the size of the experimental landscape: small (4 m^2^), medium (100 m^2^), both fragmented and unfragmented, and large fragmented (400 m^2^), and shapes to the season of sampling. Ovals are 95% confidence ellipses.

Finally, there were no differences in community composition among the four landscape sizes, with considerable overlap among species in the medium unfragmented, medium fragmented, and large fragmented treatments (Fig 7). As with nekton in Experiment 1, the small landscapes had slightly more extreme compositions, driven principally by *P*. *pugio*, *R*. *harissii*, and hermit crabs (*Pagurus* spp.) later in the year (S4 Fig). Likewise, there was strong temporal segregation in community composition, with an order of magnitude increase in the abundance of *A*. *lunata* in the fall, and similar declines in *Pagurus* spp., *P*. *pugio*, and the bruised nassa (*Nassarius vibex*) over the same period (S4 Fig).

### Experiment 2: Epifauna (Core Samples)

**Fig 8:**
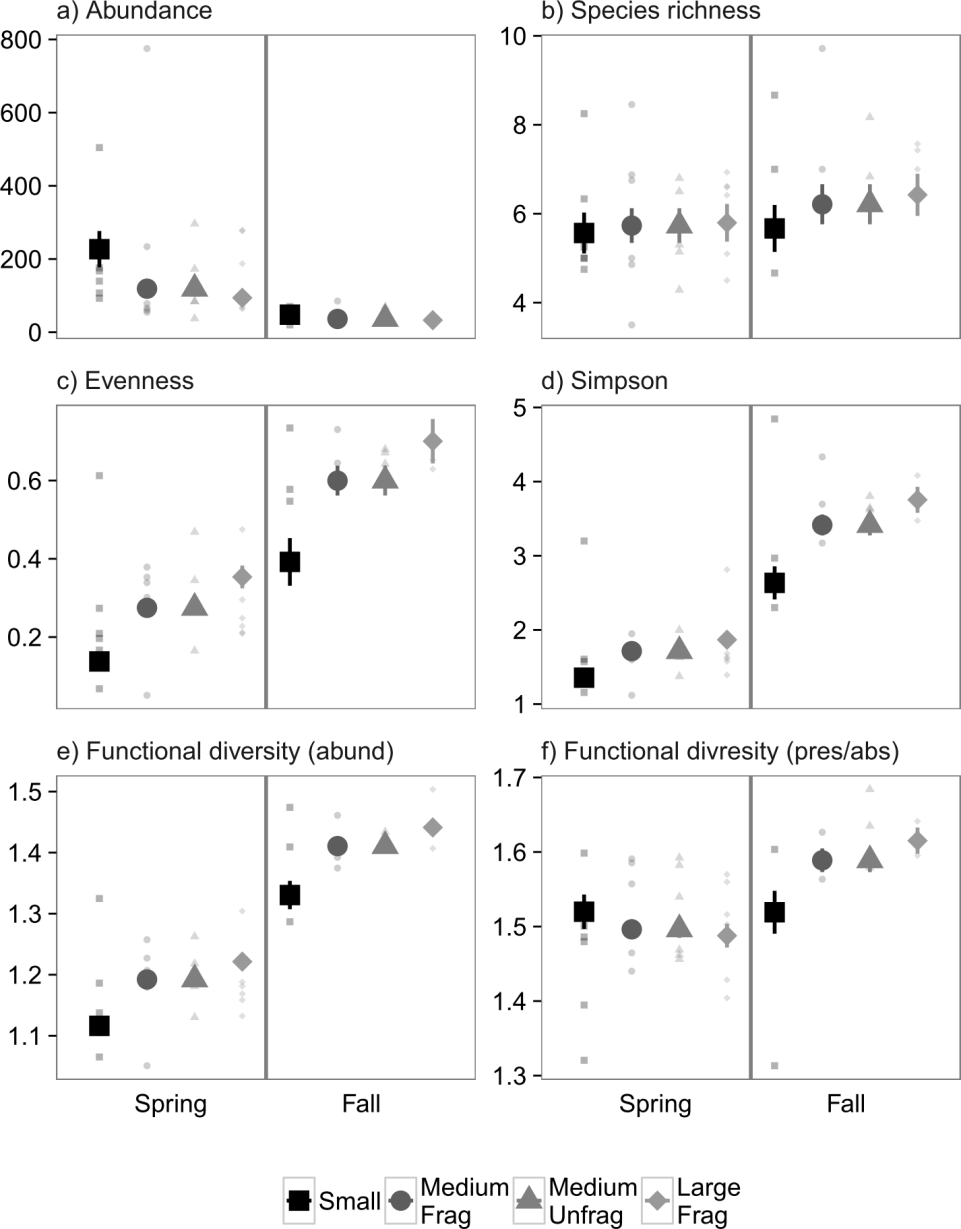
Mean values ± 1 SE m^-2^ for community properties obtained from core sampling of epifauna in Experiment 2. Colors and shapes correspond to small (4 m^2^), medium (100 m^2^), both fragmented and unfragmented, and large fragmented (400 m^2^) experimental landscapes of transplanted eelgrass. Small points correspond to replicate values. Large points are marginal means ± 1 SE estimated from generalized linear mixed effects models that account for variable sampling effort.

For core-sampled epifauna in Experiment 2, the only community metric on which landscape size had a marginally significant effect was community evenness (*P* = 0.04, Fig 8C). Parameter estimates suggested that communities are expected to become more even with increasing landscape size (β = 0.22 ± 0.10), and we emphasize no detectable differences between unfragmented medium and large fragmented landscapes, which is our truest test of fragmentation effects. The remaining community metrics showed no association with the experimental treatments (0.15 < *P* < 0.82). As in Experiment 1, community properties were strongly influenced by season, with mean abundance significantly decreasing throughout the year (*P* = 0.001, Fig 8A), and evenness (*P* = 0.006, Fig 8C), Simpson diversity (*P* = 0.002, Fig 8D), and abundance-weighted functional diversity (*P* < 0.001, Fig 8E) all increasing throughout the year. Also as in Experiment 1, there was no significant interactions between time and treatment (0.07 < *P* < 0.87), suggesting temporal changes were consistent among landscape sizes.

**Fig 9:**
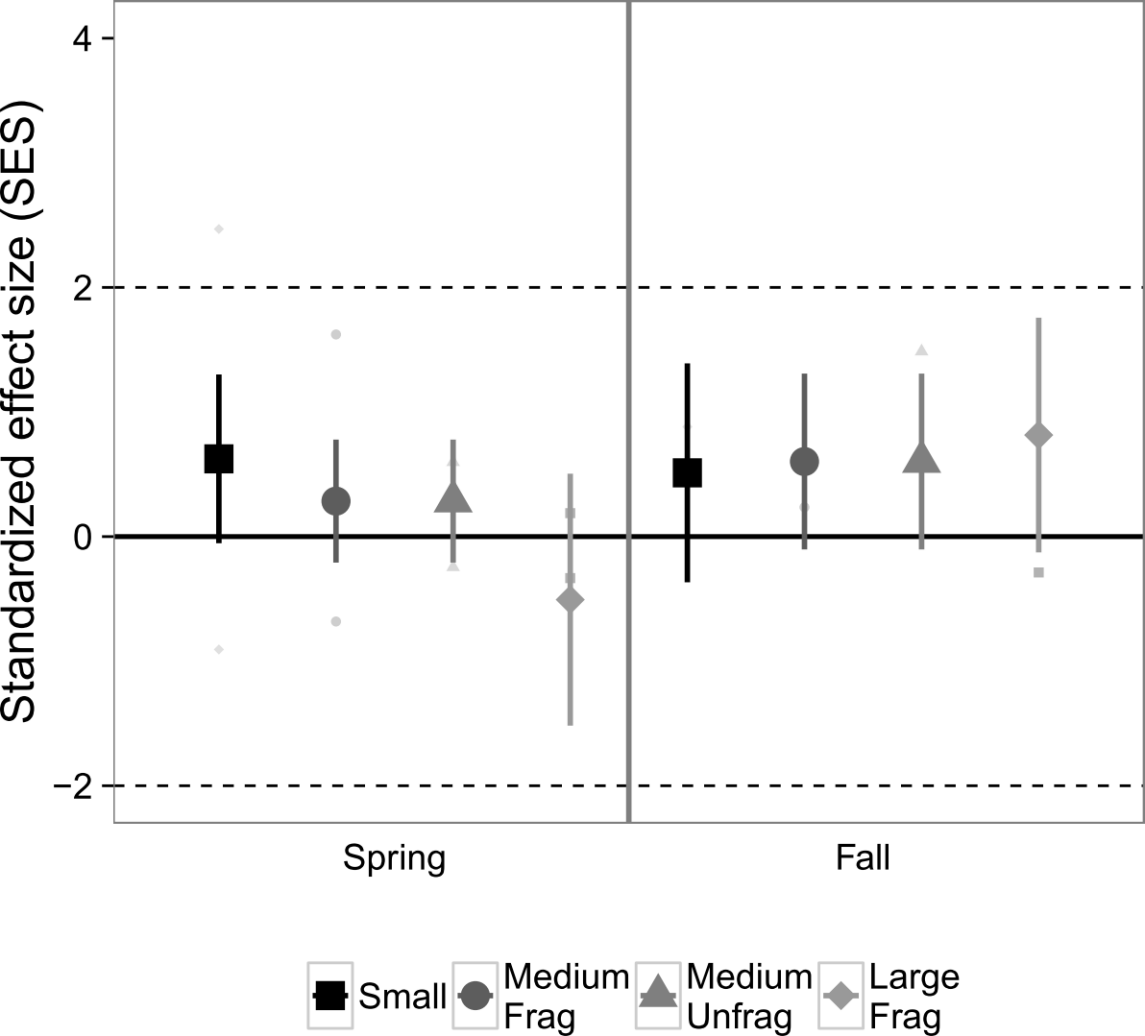
Standardized effect size (SES) from checkerboard scores, an index of epifaunal species co-occurrences in core samples from Experiment 2 across two seasons. Values >2 indicate significantly fewer associations than would be expected from chance, whereas values < -2 indicate the opposite. Values in the range [-2, 2] indicate random segregation of species in experimental replicates. Large points are marginal means ± 1 SE estimated from generalized linear mixed effects models that account for variable sampling effort. Colors correspond to the size of the experimental landscape: small (4 m^2^), medium (100 m^2^), both fragmented and unfragmented, and large fragmented (400 m^2^).

Species pairs appeared to be randomly distributed in the experimental treatments, once again indicated by the lack of standardized effect sizes falling outside of the [-2, 2] range (Fig 9), and was affected only by the number of samples taken (*P* = 0.04), based on analysis of deviance. The remainder of the predictors had no significant associations with the index (0.25 < *P* < 0.89).

**Fig 10:**
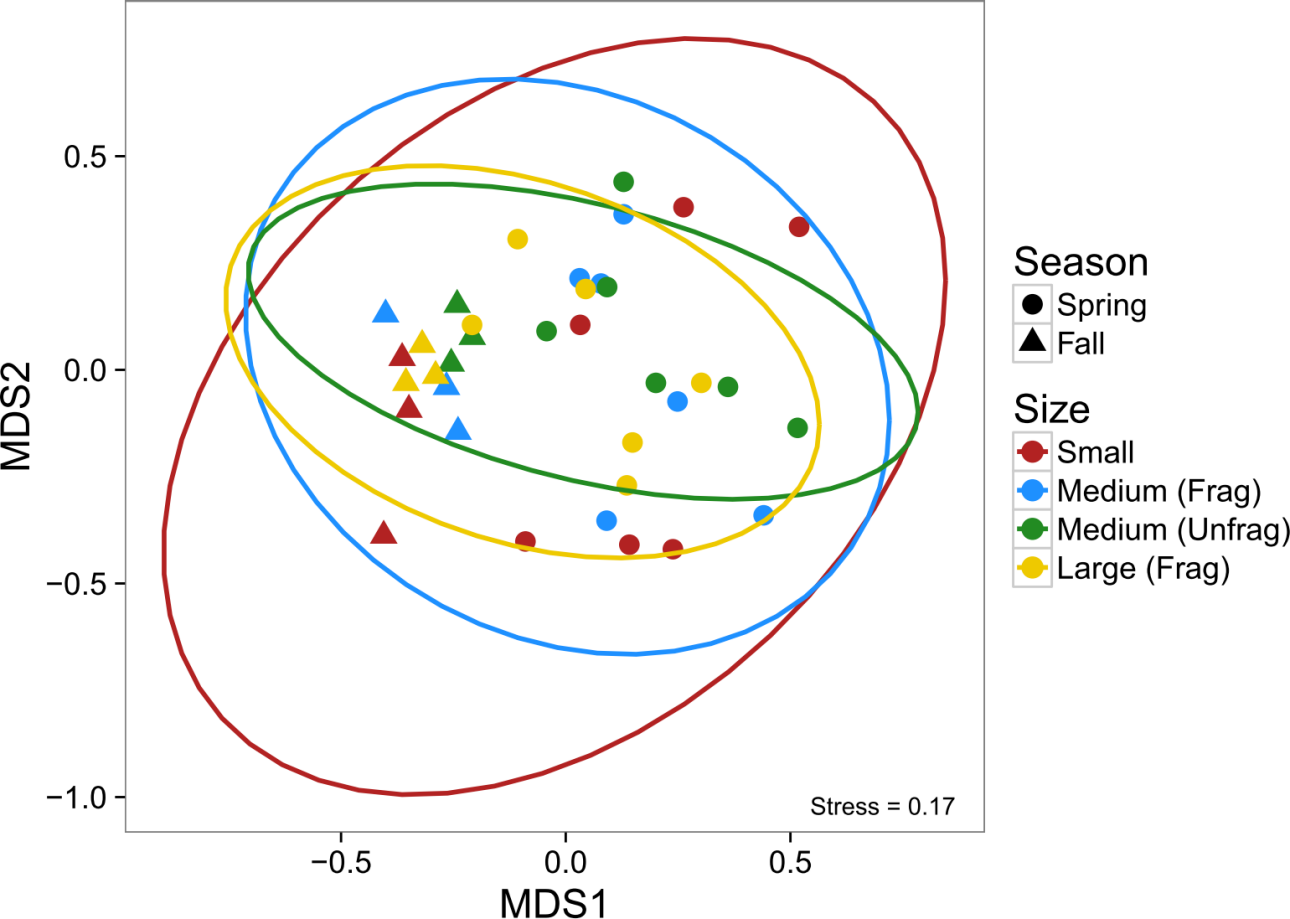
Non-metric multidimensional scaling of multivariate epifaunal community abundances from core samples in Experiment 2. Colors correspond to the size of the experimental landscape: small (4 m^2^), medium (100 m^2^), both fragmented and unfragmented, and large fragmented (400 m^2^), and shapes to the season of sampling. Ovals are 95% confidence ellipses.

Community composition was similarly unaffected by the experimental treatments, and like Experiment 1, showed marked shifts through time (Fig 10). Fall samples appear to be marked by significantly fewer isopods (*Idotea balthica*) and caprellid and gammaridean amphipods, and increases in the abundance of ampithoid amphipods and the lunar dovesnail (*Astyris lunata*) (S5 Fig).

## Discussion

In two experiments conducted in two separate years across two subestuaries, multiple seasons, and four distinct sites in the lower Chesapeake Bay, USA, we observed no influence of fragmented landscapes of varying size on faunal community abundance, diversity, composition, or competition per unit area in experimental transplants of live eelgrass shoots. We also observed some evidence to suggest that fragmentation itself has no effect on these properties, based on comparison of 100 m^2^ unfragmented and 400 m^2^ fragmented plots (controlling for total area, Experiment 2, Figs 8–10). Thus, consistent with existing evidence derived from manipulations of individual patch size, the area over which fragmentation occurs, and the total number of patches appears to have no effect on mobile animal communities on a per unit area basis in this system [6,7]. Our experiment extends previous conclusions from observational surveys and artificial substrates to manipulations of actual plants over much larger, and potentially more realistic, scales. Furthermore, our considerable spatial and temporal replication and statistical approach address many of the confounding factors proposed to have influenced previous results, including within-patch characteristics (such as shoot density), covariance in environmental characteristics, and location [6].

We suggest several potential explanations for the overall homogenization of faunal communities across landscapes of differing sizes. Foremost is the relatively high dispersal distance of marine organisms throughout the entirety of their life cycle, on the order of 10 m to 1000 km [58], which contributes to their ability to quickly colonize and exploit new habitat [7]. We note, however, that many of the organisms in our dataset are exceedingly small (median = 12 mm total length, based on literature values), and are brooders and/or direct developers (S1 Table), implying limited mobility and no or brief planktonic larval stage relative to the decapods and fishes. Despite this, prior studies have shown that these animals can disperse rapidly out of and between patches, directly or by rafting on floating material [59,60]. The success of this particular subset of species may also be attributable to their fast generation times, on the order of months to as few as three weeks [61]. Thus, increased dispersal, aided or unaided, and rapid life histories should ensure an abundant supply of individuals reaching any given patch, or even these experimental landscapes, which were distant from existing eelgrass beds. Indeed, a recent study conducted in nearby coastal systems has shown that animal communities recruiting to newly restored eelgrass beds were indistinguishable from those in existing mature beds in fewer than two years [40]. Our results suggest that the recruitment can occur even more rapidly, based on surveys of nearby natural beds conducted concurrent with Experiment 1 (S3 Fig), and previous studies have shown similar results on the scale of weeks [11].

Further, we did not observe any evidence for an ‘extinction debt’ in increasingly large habitats [17]. Instead, diversity–and by extension species composition–appeared to be primarily under seasonal control and generally increased throughout the year (Figs 2 and 5), an observation that is consistent with other long-term studies in this system [62,63]. It may be that temporal turnover is so rapid as to outpace treatment effects: species emigrate via natural processes before they have a chance to go locally extinct. The drivers of faunal diversity and composition through time are not completely understood, but may have several, non-mutually exclusive causes. First, the Chesapeake Bay is an environmentally dynamic estuary, with temperature ranges exceeding 25°C annually [62]. Varying physiological tolerances may generate turnover as cold-tolerant species, such as gammaridean amphipods [64], are replaced by warm-affinity ones as the year progresses [62]. This hypothesis has interesting implications for climate change, which is expected to warm the Chesapeake Bay 2–6°C over the next century [65]. Such warming may shift the composition and relative dominance of seagrass faunal communities, as has been seen farther south [66], with the potential to increase the timing and severity of fragmentation effects. Indeed, a recent meta-analysis of over 1,300 papers spanning aquatic and terrestrial realms has revealed maximum temperature as the single strongest modifier of the negative effects of habitat loss and fragmentation on species abundance and diversity [67]. Future studies should keep this hypothesis in mind when devising fragmentation experiments to maximize their relevancy.

Second, many of the nekton species in the Chesapeake Bay have complex life histories, migrating into shallow waters in the summer months to feed and spawn, and into deeper channels or offshore as the water cools, in the case of pipefishes [68]. Others recruit into the bay as juveniles and leave as adults, such as blue crabs [69]. Together, these biological and physiological factors should reduce competitive interactions and the potential for exclusion by staggering functionally redundant species through time [63], which is likely why we failed to observe any signal of competitive structuring in these communities (Figs 4, 7 and 10), in contrast to previous studies [70]. While other seagrass systems may not be as extreme as the Chesapeake Bay, they often exhibit similar albeit less exaggerated seasonal trends [71–73], and thus temporal dynamics overshadowing fragmentation effects, at least on the scales tested here and elsewhere, may be a complementary explanation for why we generally fail to see a uniform response to fragmentation in seagrass beds [6,7].

As others have noted, increased mobility and dispersal of estuarine fauna should yield greater encounter rates for smaller patches, by virtue of their high edge to interior ratio, increasing abundance and diversity over small areas [14]. That we observed no change in these responses with landscape size suggests that propagule supply is not the only factor driving community composition. In addition to environmental temporal drivers, one additional alternative is resource control. A recent long-term analysis of seagrass beds in the Chesapeake Bay revealed that consumers, principally grazing epifauna, are limited by both habitat and food resources, particularly in the fall [63], a conclusion that has been reached in other systems [70] and experimental manipulations [74]. Since habitat was standardized in this experiment, it stands to reason that constraints on food–epiphytic microalgae growing on the blades of eelgrass–may have set a cap on the abundance and diversity of grazers per unit area. Such ‘resource ceilings’ are a well-described phenomenon in seagrass epifauna [75,76], and could explain the consistent community responses across the different landscape sizes. In turn, primary producer constraints on epifauna should also constrain prey availability for larger, more mobile predators, in a classic example of bottom-up forcing, leading to the generally consistent null response of more mobile nekton as well. It is worth noting that the mesopredators considered in this study are themselves food for larger, more mobile predators. Thus, top-down control may be a prominent driver of trends in nekton diversity, with lower values in the fall after many predators have emigrated into the Chesapeake Bay (Fig 5B–5F) [68]. Top-down control may also explain the lack of competition observed in the experimental plots, as the role of predators in reducing competitive interactions is a well-documented phenomenon [77].

A recent paper emphasized that focusing on overall biodiversity ignores the potentially contrasting responses of individual species to fragmentation, in particular whether they are habitat generalists or specialists [78]. This outcome is not likely to hold for our study, since all of the animals we observed are distributed widely along the eastern coast of the US and in a variety of habitats, including salt marshes, oyster reefs, and mud flats [68,79], and therefore could not be considered specialists. Accordingly, there were not significant differences in the abundance of individual species among the different landscape sizes (based on overlap of error bars, S3–S5 Figs), with the notable exception of *P*. *pugio*, which was consistently more abundant in small patches, particularly in the fall (S3 and S5 Figs). This species is generally redundant in terms of trophic ecology and body size with many of the other organisms in our surveys (S1 Table), but has the somewhat unique trait of brooding eggs from which emerge planktonic larvae [80], as opposed to the peracarid crustaceans, which also brood their eggs but which hatch into miniature adults with no external larval stage [81]. This trait, coupled with recruits from a spring spawn [82], may explain the surplus of adult *P*. *pugio* in small plots later in the year, where their settling larvae would have experienced greater interception rates with edge habitat, as proposed by [11]. The relative functional uniqueness of *P*. *pugio* may also explain the trend of decreasing functional diversity with increasing landscape size in E2 (Fig 5E and 5F). Prior studies have also observed higher densities of *Palaemonetes* spp. in small patches, suggesting alternatively that this trend may reflect more effective foraging in edge habitats [83].

Because we did not modify, revert, or otherwise alter the density or configuration of the fragmented landscape, we are unable to make any statements about how the process of fragmentation *stricto sensu* influenced faunal communities, only the outcome of this process over the longer term. Several prior studies that have expressly looked at the fragmentation process, however, have not found differences in community properties in response to fragmentation after longer time periods versus those observed over very short timescales while habitats were being fragmented [12,84]. Thus we feel our results likely reflect what likely occurred immediately post-fragmentation, although future manipulations could sample during this period to generalize findings from previous studies conducted elsewhere. We also did not impede the natural expansion of the grass to maintain the experimental treatments (e.g, by weeding), and still did not see significant differences in eelgrass density over the course of either experiment. This finding suggests that our study is reflective of how fauna would respond to fragmentation in natural beds, which, unlike artificial substrates, are capable of both sexual recruitment and clonal growth. It is worth noting that these most of these plots did eventually fill in [35], although we did not continue to sample faunal communities following these experiments.

If many hypotheses explaining fragmentation effects have been structured around the amount of edge habitat, why did we not test for statistical effects of edge area? Unfortunately, our experimental design confounds total area with edge area: Pearson correlations between total area and edge area range from *r* = 0.995–1.00 in Experiment 1, and *r* = 0.73–0.98 in Experiment 2 (slightly lower on account of the medium unfragmented treatment). Thus, the high degree of collinearity precludes us from independently testing both, and since the experiments were initially designed to test the effects of total area [33,34], we have decided to present that as the primary treatment effect in our analysis. We suggest future explorations of the topic of landscape fragmentation vary total and edge area orthogonally, for instance, by altering the degree of fragmentation or the shape and configuration of the fragments [85]. To that end, we also recommend that investigators vary the number of replicates to combat the issue of uneven replication across landscape sizes. A sampling regime that replicates small patches more highly than large patches would offset the higher number of samples necessitated to thoroughly sample large patches, and permit the use of the raw vs. summarized data, increasing statistical power and therefore the ability to discriminate treatment effects.

One of the enduring debates in conservation biology is “single large or several small,” referring to the amount and configuration of habitat set aside or created to protect and maintain existing diversity. Others have proposed that several small seagrass beds may be equally or more effective for conserving the diversity of macroinvertebrates and fishes [14], and infaunal assemblages [21]. Our results lend additional support to the idea that small patches can support equivalent diversity of species and their functional traits across multiple sites, years, and trophic levels. This conclusion has implications for seagrass conservation and restoration, suggesting that small patches–which are more feasible and cost-effective to construct or protect–may provide equivalent services, at least in terms of animal communities. However, it is important to keep in mind that there exist trade-offs between patch size and retention, where patches that are too small are unable to withstand environmental forcing and survive to reproduce [86]. We also note that our study, while one of the most comprehensive and realistic, still has relatively low density of eelgrass relative to natural beds, and does not consider many additional factors likely operating in natural systems, including repeated disturbance and fragmentation, greater ranges of environmental variability, and other human impacts, such as pollution or fishing, that are known to modify faunal communities. Thus, while our study appears to reinforce general conclusions from a substantial body of work relating to seagrass fragmentation effects [6,7], we caution that fragmentation may have more extreme consequences in other seagrass systems subject to different combinations of stressors, or with a different suite of animals that have the potential to influence either top-down or bottom-up characteristics [87].

Nevertheless, we show that the primary mechanism by which seagrass fragmentation may affect faunal communities in this estuary is not likely to be via changes in the inherent structure of the remaining communities, but rather by reducing the total habitat area and thereby the total potential productivity of the system, consistent with emerging consensus about fragmentation effects in seagrass systems in general [6]. Instead, the mobile, fast-reproducing, and generally omnipresent animal community is keenly responsive to the presence of habitat, rapidly colonizing newly established seagrass with little regard for its distribution in the seascape. Thus, a greater concern should be the loss of seagrasses altogether [3], which will reduce animal abundance and diversity through the removal of their essential habitat, rather than fragmentation.

## Supporting Information

S1 Fig Percent cover (top row) assessed using quadrats and shoot density (shoots m^-2^, bottom row) assessed using ring counts for three sites in Experiment 1 (CG = site 1, York River, MS = site 2, James River, SB = site 3, James River) in two seasons, and for each experimental landscape size: small (4 m^2^), medium (100 m^2^), and large (400 m^2^). (TIF)

S2 Fig Percent cover (top row) assessed using quadrats and shoot dry weight (g, bottom row) assessed from core samples for two sites in Experiment 2 (York River and James River) in two seasons, and for each experimental landscape size: small (4 m^2^), medium (100 m^2^), both fragmented and unfragmented, and large fragmented (400 m^2^). (TIF)

S3 Fig Log_10_-transformed mean abundances ± 1 SE for species obtained during suction samples in Experiment 1 for each sampling dates in 1997 and for each experimental landscape size: small (4 m^2^), medium (100 m^2^), and large (400 m^2^). Control refers to an adjacent natural eelgrass bed, sampled to determine whether natural faunal communities resembled ones recruiting to the experimental transplants.
(TIF)

S4 Fig Log_10_-transformed mean abundances ± 1 SE for species obtained during suction samples in Experiment 2 for the two samples dates in 1999 and for each experimental landscape size: small (4 m^2^), medium (100 m^2^), both fragmented and unfragmented, and large fragmented (400 m^2^). (TIF)

S5 Fig Log_10_-transformed mean abundances ± 1 SE for species obtained during core samples in Experiment 2 for the two samples dates in 1999 and for each experimental landscape size: small (4 m^2^), medium (100 m^2^), both fragmented and unfragmented, and large fragmented (400 m^2^). (TIF)

S1 File R code and data (CSV) used to conduct all analyses and produce all figures. (ZIP)

S1 Table Functional traits used in the analysis of functional diversity, their units, and ecological interpretations. (PDF)

S2 Table Functional traits used in the analysis. (XLSX)
