# Supplementary figures and images for: Faunal Communities Are Invariant to Fragmentation in Experimental Seagrass Landscapes
Source: PLoS One. 2016 May 31;11(5):e0156550. doi: 10.1371/journal.pone.0156550 (PMC4887026; doi:10.1371/journal.pone.0156550)

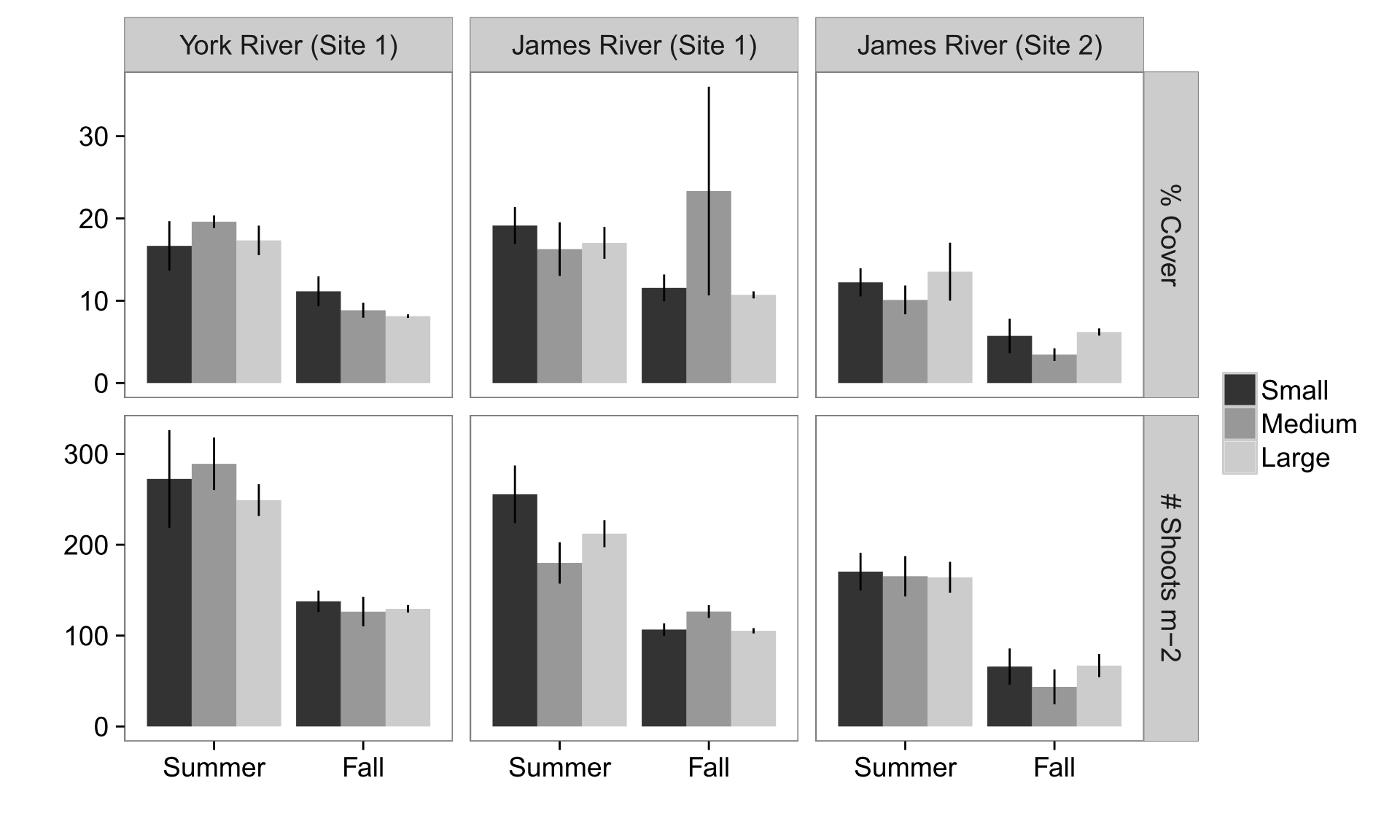

Supplement: S1 Fig — (TIF) [file pone.0156550.s001.tif]

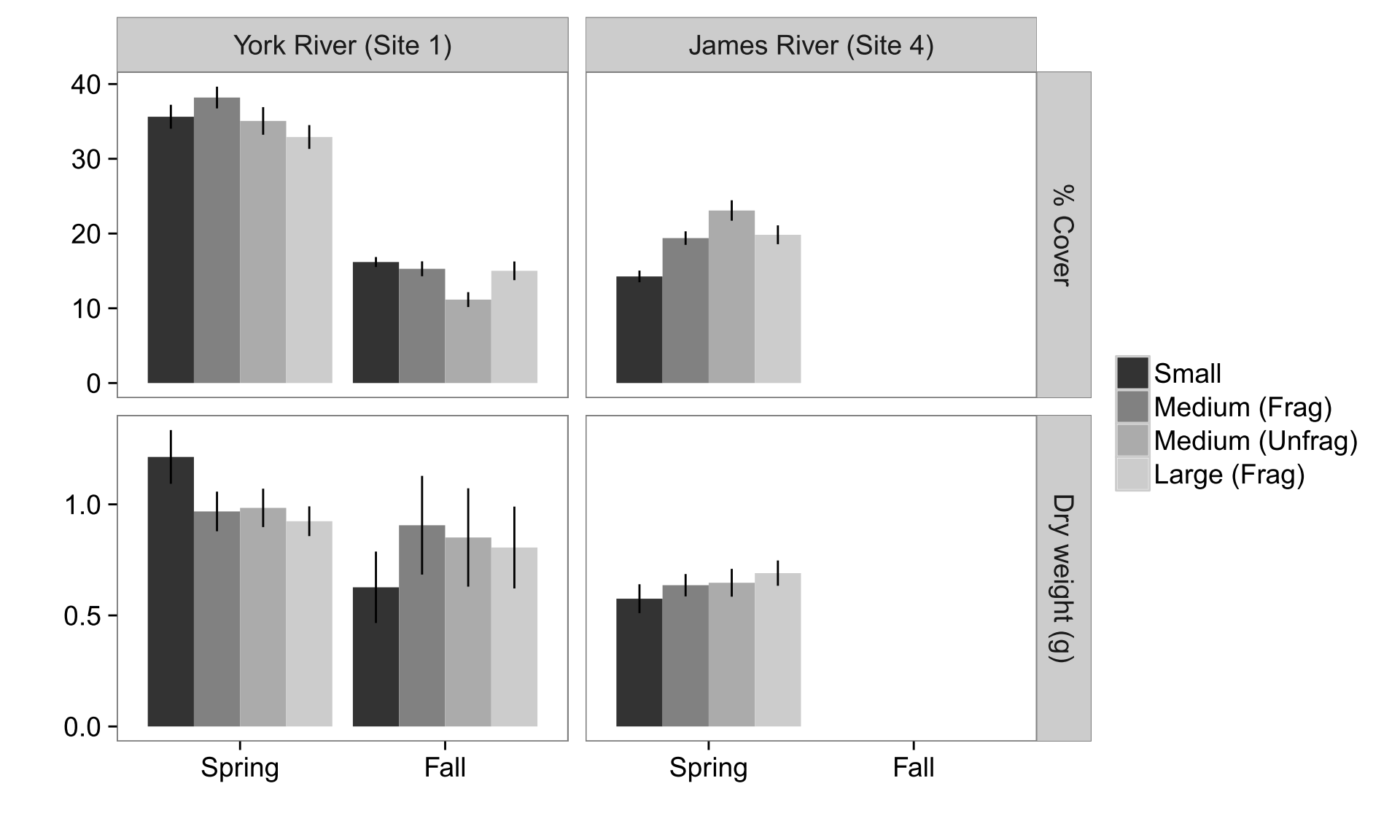

Supplement: S2 Fig — (TIF) [file pone.0156550.s002.tif]

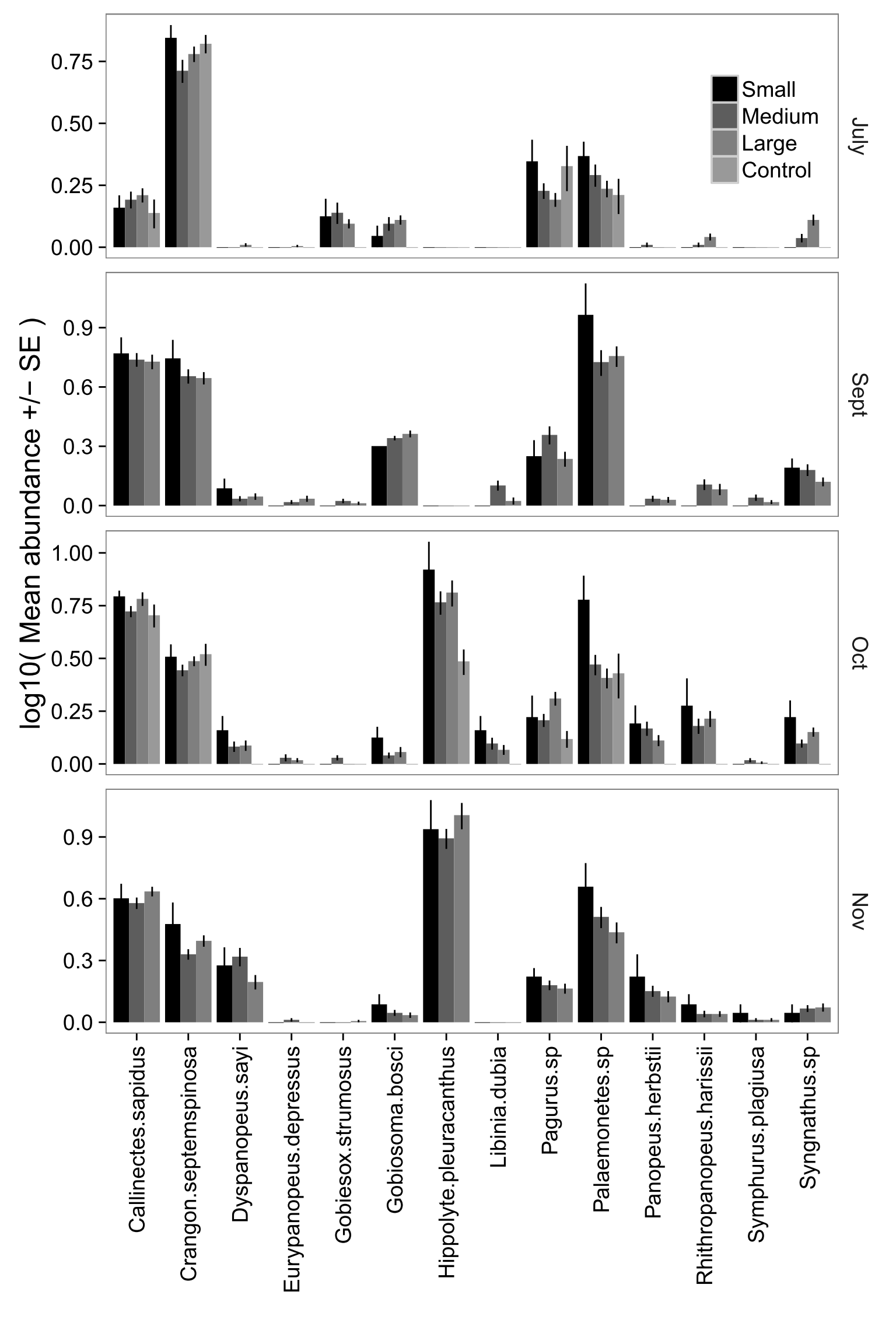

Supplement: S3 Fig — Control refers to an adjacent natural eelgrass bed, sampled to determine whether natural faunal communities resembled ones recruiting to the experimental transplants. (TIF) [file pone.0156550.s003.tif]

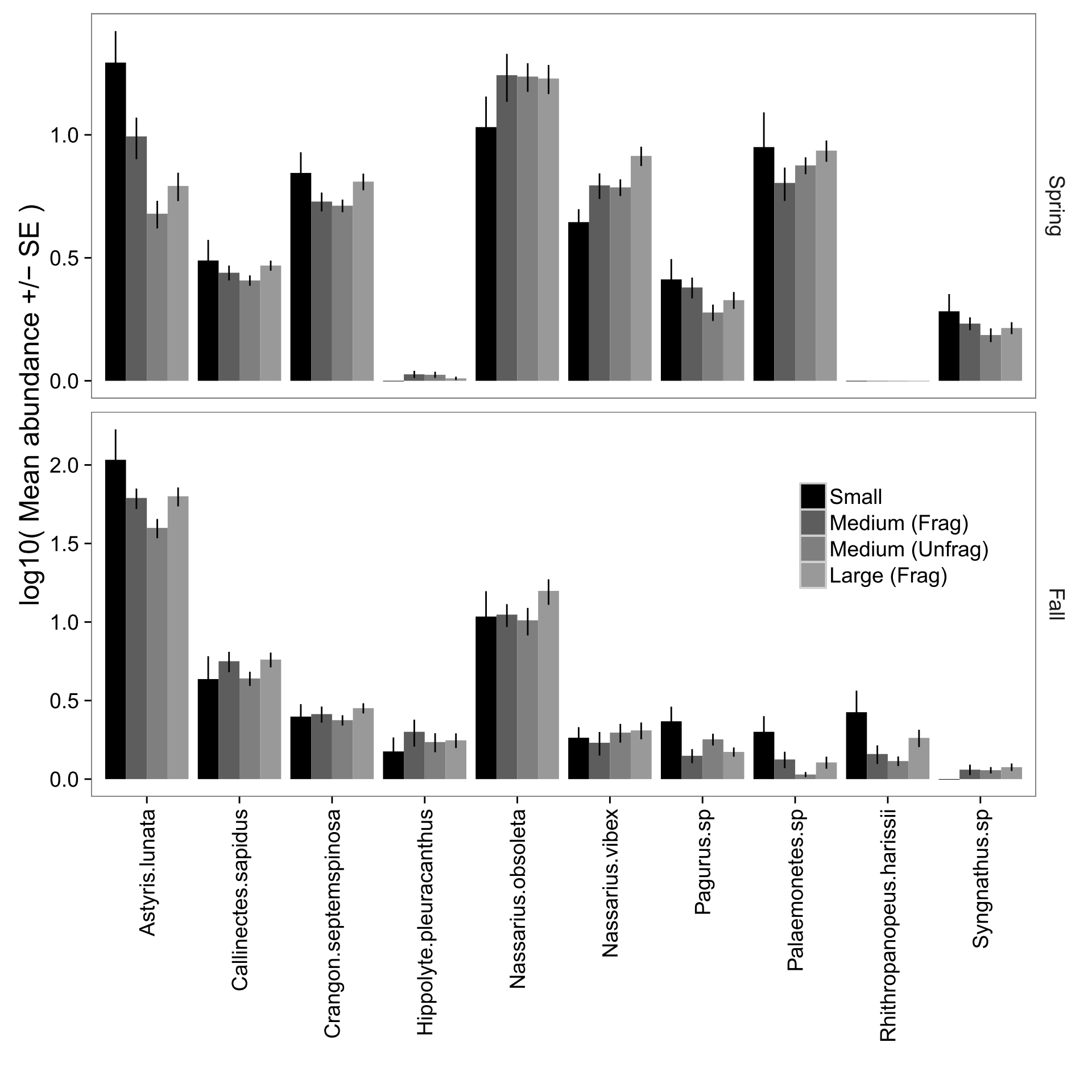

Supplement: S4 Fig — (TIF) [file pone.0156550.s004.tif]

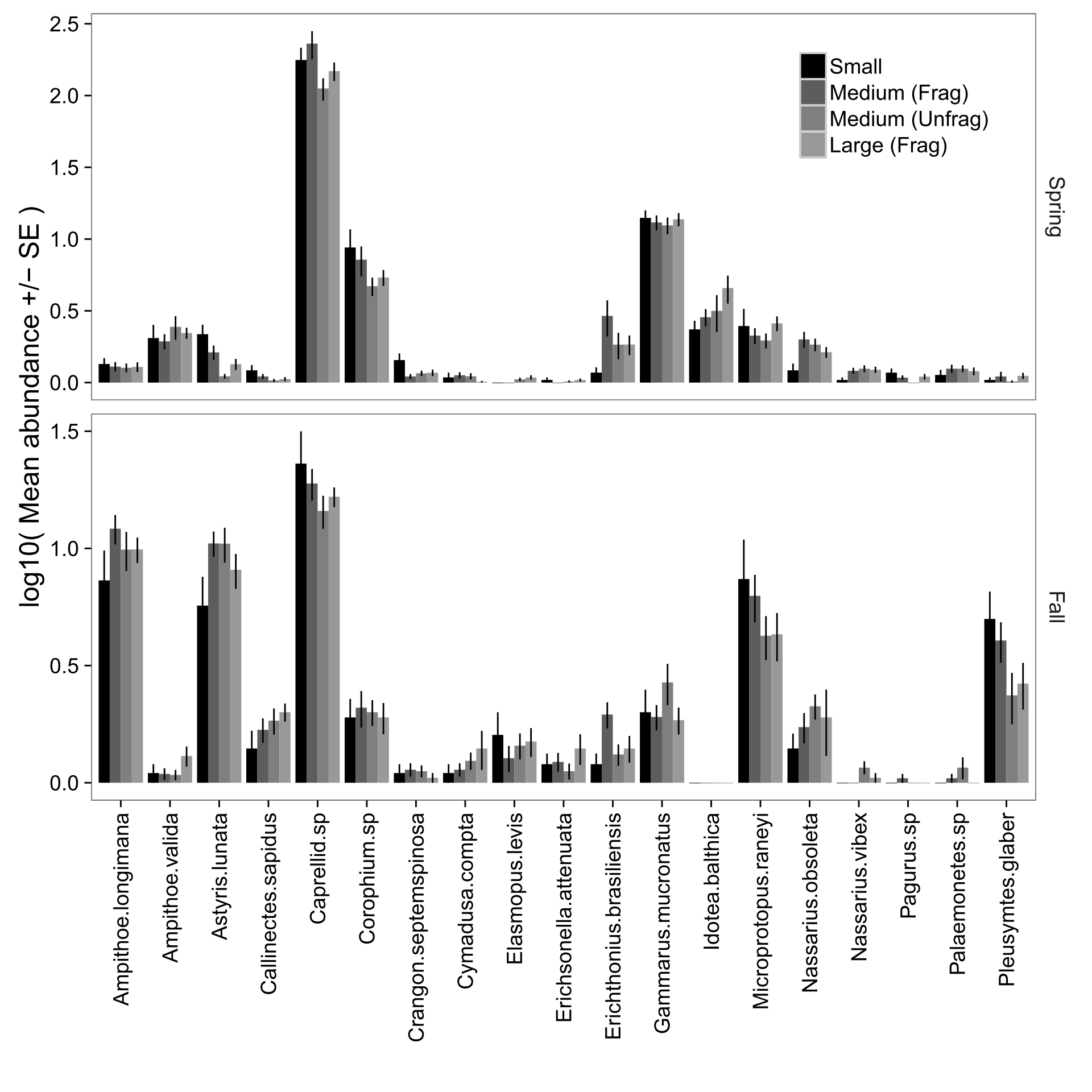

Supplement: S5 Fig — (TIF) [file pone.0156550.s005.tif]
