# Supplementary material for: Faunal Communities Are Invariant to Fragmentation in Experimental Seagrass Landscapes
Source: PLoS One. 2016 May 31;11(5):e0156550. doi: 10.1371/journal.pone.0156550 (PMC4887026; doi:10.1371/journal.pone.0156550)
Supplement: S1 Table — (PDF) [file pone.0156550.s007.pdf]

S1 Table: Functional traits used in the analysis of functional diversity, their units, and ecological interpretations.

Trait	Description	Units	Functional Interpretation
Exoskeleton material	The primary material used in forming the exoskeleton	Calcium carbonate, chitin	Defense and competition
Trophic group	The primary trophic guild of the organism	Detritivore, filter feeder, grazer, omnivore, predator	Resource partitioning
Specific diet	The general group that forms the majority of the diet based on published analyses of diet preferences and/or gut contents	Benthic microalgae, bivalves, crustaceans, detritus, epibionts, epiphytic microalgae, hydroids, macroalgae, microalgae, polychaetes, sponges	Resource partitioning
Maximum length	The largest recorded size of the organism	Continuous, in mm	Habitat use and partitioning, resource partitioning
Mobility	The general state of activity of the organism	Crawling, free-swimming, tube-building	Habitat use and partitioning, colonization potential
Egg retention	Whether the organism retains its eggs or releases them into the water column	Brooding, external release	Colonization and dispersal potential
Development mode	Whether the organism has a larval stage, and, if so, if that larvae disperse in the plankton	Direct, larval (non-planktonic), larval (planktonic)	Colonization and dispersal potential
